# High-frequency shock wave lithotripsy: stone comminution and evaluation of renal parenchyma injury in a porcine ex-vivo model

**DOI:** 10.1007/s00345-023-04441-9

**Published:** 2023-06-07

**Authors:** Marie-Claire Rassweiler-Seyfried, Jürgen Mayer, Cedric Goldenstedt, Rafael Storz, Ernst Marlinghaus, Gerold Heine, Peter Alken, Jens Jochen Rassweiler

**Affiliations:** 1 Department of Urology and Urosurgery, Medical Faculty Mannheim, University Medical Center Mannheim, University of Heidelberg, Theodor-Kutzer-Ufer 1-3, Mannheim, Germany; 2 Storz-Medical AG, Taegerwilen, Switzerland; 3 Department for Urology and Andrology, Danube Private University, Krems, Austria

**Keywords:** SWL, Stone fragmentation, Stone comminution, High-frequency shock waves, Burst-SWL, Shock-wave induced renal trauma

## Abstract

**Background:**

The electrohydraulic high-frequency shock wave (Storz Medical, Taegerwilen, Switzerland) is a new way to create small fragments with frequencies up to 100 Hertz (Hz). This study evaluated the efficacy and safety of this method in a stone and porcine model.

**Materials and methods:**

BEGO stones were put in a condom in a specifically designed fixture treated with different modulations to see stone comminution. Standardized ex vivo porcine model with perfused kidneys with 26 upper and lower poles of 15 kidneys was treated with the following modulations: voltage 16–24 kV, capacitor 12 nF and frequency up to 100 Hz. 2000–20,000 shock waves were applied to each pole. The kidneys were perfused with barium sulfate solution (BaSO4) and x-ray was performed to quantify the lesions using pixel volumetry.

**Results:**

There was no correlation between the number of shock waves and the powdering degree or the applied Energy and the grade of pulverization in the stone model. Regarding the perfused kidney model, the number of shock waves, applied voltage and frequency had no direct correlation with the occurrence of parenchymal lesions The detected lesions of the renal parenchyma were minimal, technical parameters had no significant impact and the lesions did not differ from the results of former experiments using 1–1.5 Hz in the same model.

**Conclusions:**

High-frequency shock wave lithotripsy can produce small stone fragments to pass in a very short time. The injury to the renal parenchyma is comparable to the results of the conventional SWL using 1–1.5 Hz.

## Introduction

The use of extracorporeal shock wave lithotripsy (SWL) has decreased in recent decades due to changing indications and technological developments [1–3]. Although SWL still offers the least invasive treatment for urolithiasis, more patients are now treated endoscopically. Endourologic stone treatment achieves fine fragmentation using the dusting mode of the Holmium-laser or Thulium-fibre laser, practically eliminating the risk of a steinstrasse. Therefore, shock wave lithotripsy can only survive if the quality of fragmentation can be significantly improved [4, 5].

The ideal SWL produces small fragments of kidney stones quickly without causing any significant injury to the renal parenchyma. Recent technological developments, such as Burst-wave lithotripsy (BWL), can achieve such fine pulverization of stones [6, 7]. Burst waves are modified ultrasound waves applied at a high frequency (390 kHz) for a short time (59 microseconds). In previous experiments, BWL achieved much finer fragmentation in a shorter period of time [6]. Animal studies showed only minimal lesions in the porcine kidney. First clinical studies are promising but also highlight some shortcomings [7, 8]. Upper pole stones or stones that are too small, too deep, or obstructed by a rib or bowel, which are therefore not visible using ultrasound, cannot be treated with BWL [7–10].

The idea to use high-frequency shockwaves under continuous application has existed since the end of the last century but has never been realized in a defined clinical experimental set-up [11]. The EDAP LT01 device allowed for the application of high-frequency shock waves up to 100 shocks per second, but clinically most treatments were administered at a frequency of 1.25 or 2.5 Hz. In 1988, Delius et al. published several studies on the application of high-frequency shock waves using the 40 nF Generator at 20 kV (Dornier HM1). They described significant hemorrhage in the outer medulla of the canine kidney. They thought that this was not attributed to a direct effect of the shock wave but due to the induced cavitation [12].

The modified electrohydraulic high-frequency shock wave source uses a 12 nF generator (Storz Medical, Taegerwilen, Switzerland) as a new concept to create small fragments with frequencies up to 100 Hertz (Hz). In this paper, we present the first in-vitro results concerning the effect on the fragmentation of test stones, as well as the impact on renal tissue in a standardized model of perfused porcine kidneys.

## Material and methods

### Experimental lithotripter

**Fig. 1:**
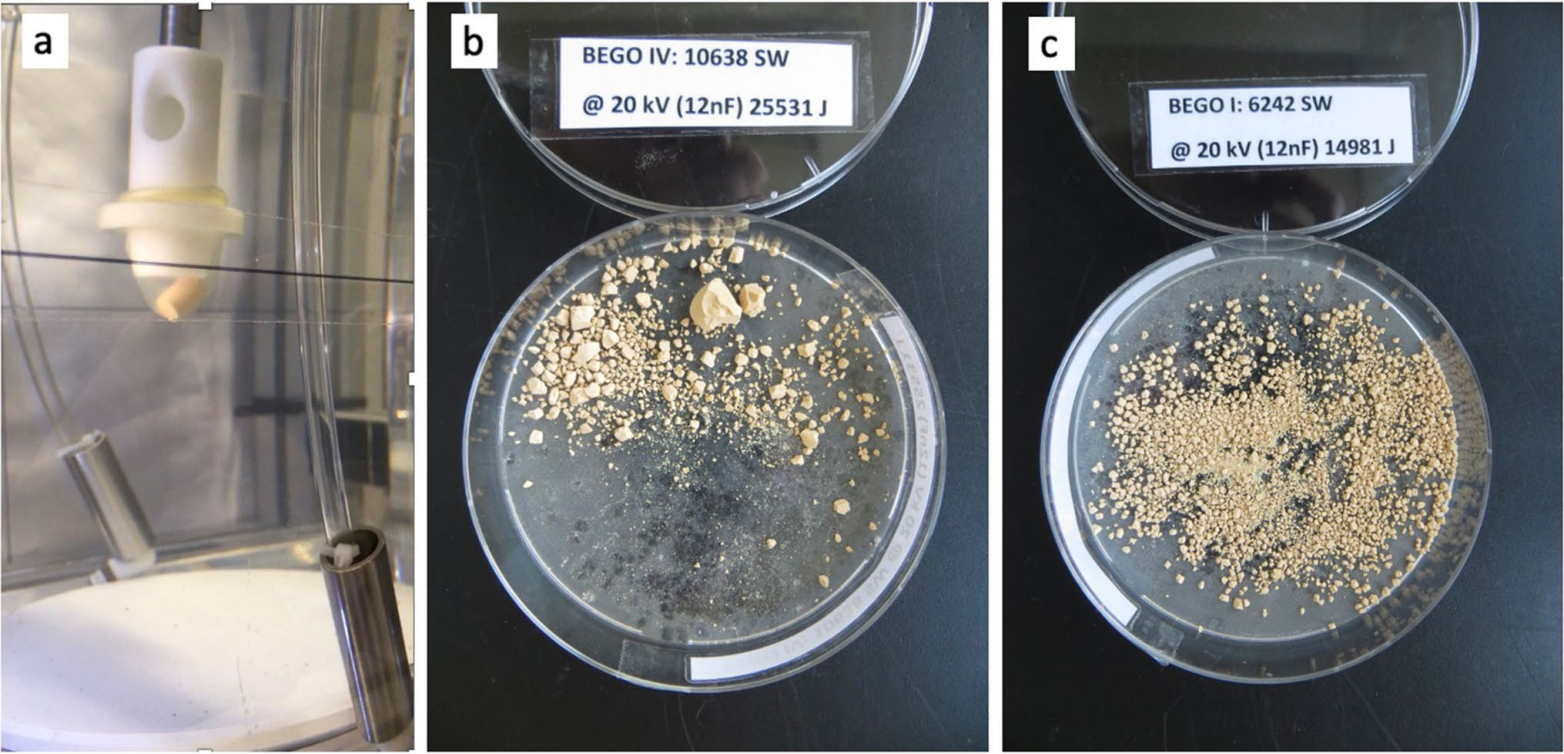
In-vitro model for stone-disintegration. **a** Electrohydraulic 12 nF-Generator for generation of high-frequency shock waves. Bego-stones are located in a condom with mechanical adjustment. **b** Incomplete fragmentation with larger residual fragments (++) using 10,638 shock wave at 20 kV in 13 burst applications (210–1000 SW) with three re-adjustments. **c** Complete fine fragmentation (+++) using 6242 shock waves at 20 kV in 8 burst applications (556–1000) with two re-adjustments

The experimental lithotripter consisted of electrohydraulic device with an underwater spark electrode (F1) with an ellipsoid reflector. A 12 nF generator was used enabling the application of frequencies up to 100 Hz (Fig. 1a).

### Stone model

For the tests of stone fragmentation, BegoStones made of commercially available super-hard plaster were used. Liu and Zhong characterized them in comparison to human kidney stones comparable to calcium-oxalate monohydrate [13]. Nine BegoStones were submerged in deionized water 72 h prior to the experiment. The stones were all shaped like cylinders in a height of 10 mm and 5 mm wide. They were put in a condom filled with degassed water in a specifiallyc designed fixture. Focusing of the stones was done optically based on the calculated F2 in the tank filled with degassed water at 22 °C (Fig. 1a). The stones were then exposed to different modulations to see the stone comminution varying from 16 to 20 kV at a frequency of 100 Hz. The shocks were applied in 2–12 bursts of 400–1000 impulses with readjustment of the shock wave source (Table 1). Endpoint represented fine fragmentation of the test stone in fragments smaller than 1 mm (Fig. 1b).

**Table 1:** Technical data and degree of fragmentation using BEGO-Stone model

N	Generator capacity (nF)	Generator voltage (kV)	Frequency (Hz)	Number of shocks (*n*)	SW-energy (J)	Fragmentation
2	12	16	100	10.242	15,575	++
2	12	18	100	9.933	21.566	+++
3	12	20	100	8.869	19.363	+++
2	12	20	100	7.563	18.151	++

+ insufficient fragmentation; ++ fine fragmentation with larger fragments; +++ fine fragmentation

### Model of isolated perfused kidney

We used a standardized in-vitro porcine model with a perfused kidney, according to previous studies evaluating the impact of shock waves of different sources and intensities [14].

**Fig. 2:**
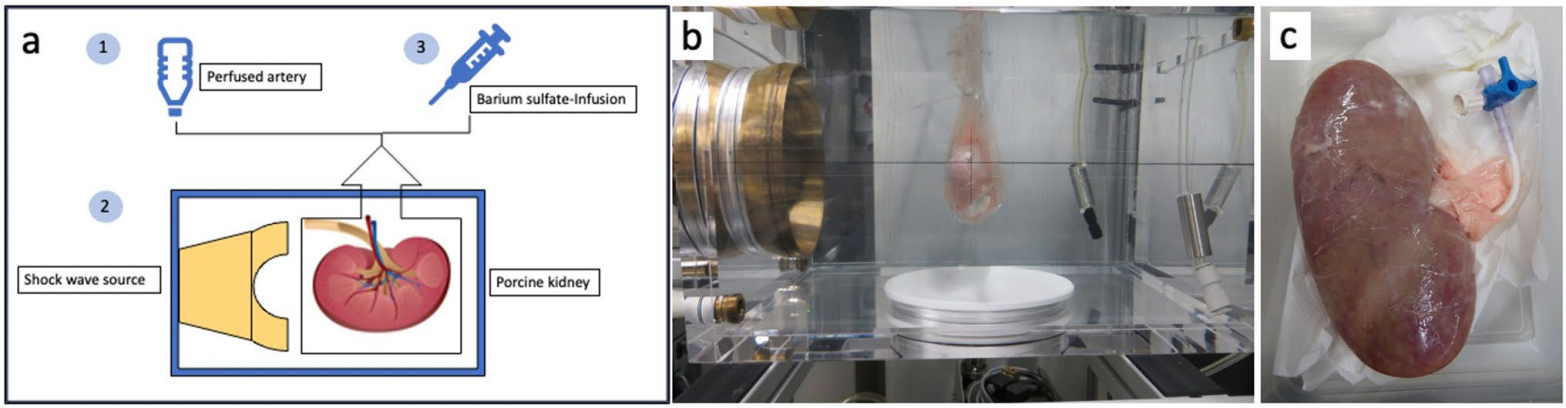
Ex-vivo model of the perfused porcine kidney. **a** Schematic drawing of the model with the main components. **b** Adjustment of the kidney in a bag filled with degassed water. **c** Perfusion of the treated kidney with barium-sulfate-solution

Immediately after slaughtering of the pigs, the kidneys were perfused using Tyrode’s solution until the eluate from the vein was clear. Afterwards, the porcine kidney was cannulated and perfused (37 °C, oxygenated solution) during shock wave application (Fig. 2a–c).

**Fig. 3:**
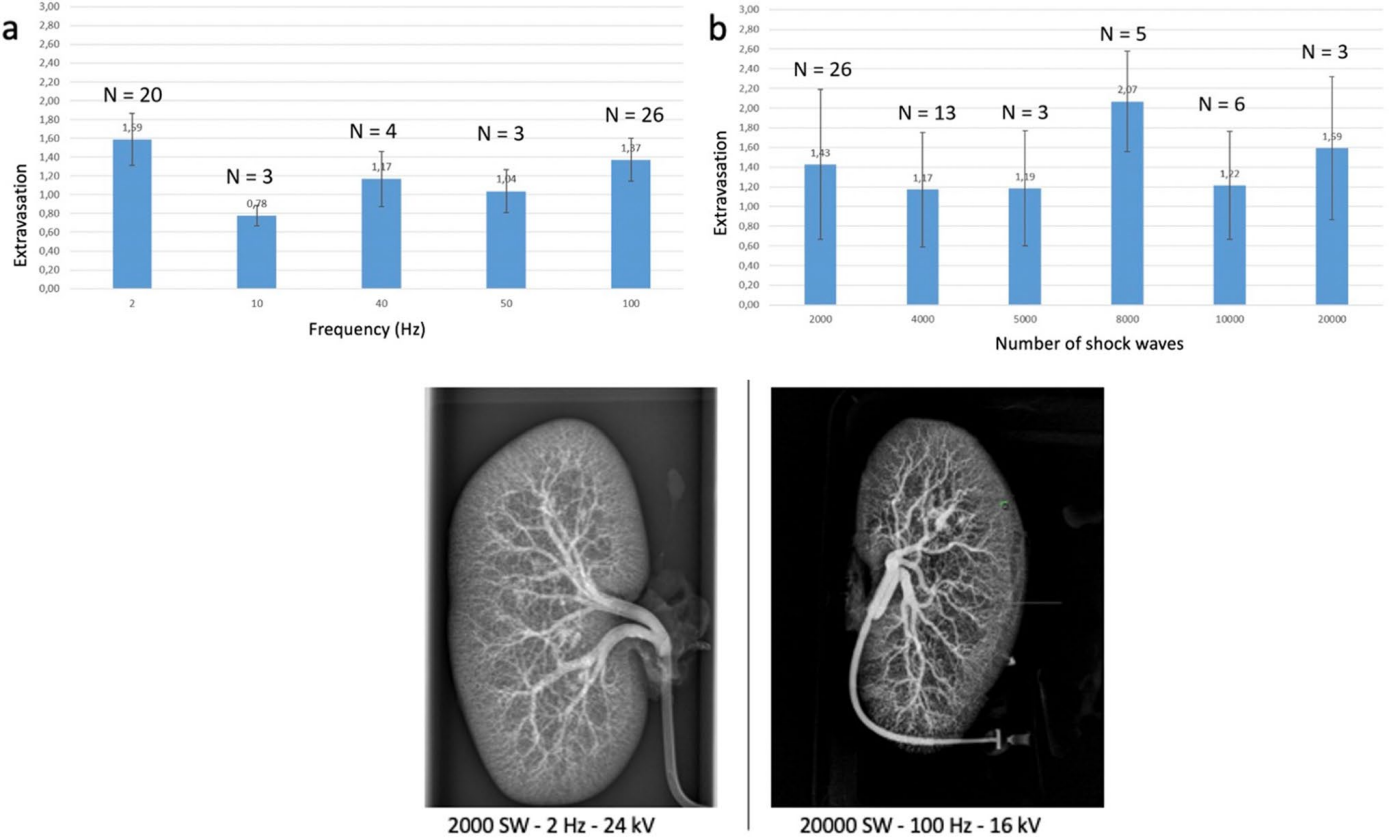
Comparison of renal lesion induced by high-frequency SWL versus classical SWL. Left: Classical SWL (44 nF generator) using 2000 SW at 24 kV with 2 Hz—only grade 1 lesion at upper and lower pole. Right: High-frequency SWL (12 nF generator) using 20,000 SW at 16 kV with 100 Hz—grade 1-lesion at upper and lower pole. Impact of technical parameters on renal lesions in the ex-vivo model of porcine kidney. **a** Frequency (Hz). **b** Number of impulses (SW)

26 upper and lower poles of 15 kidneys were treated with the following modulations: voltage 16–24 kV, capacitor 12 nF and frequency up to 100 Hz. 2000–20,000 shock waves were applied to each kidney pole. As controls, 20 upper and lower poles of 10 kidneys were treated with parameters of standard lithotripsy (44 nF capacitor, 2 Hz, 16–24 kV, 2000 SW). Subsequently, all kidneys were perfused with barium sulfate solution (BaSO4) and x-ray was performed (Fig. 3). The vascular lesions demonstrated by the BaSO4 were quantified using pixel volumetry. Pixel volumetry is a quantitative imaging technique, which was used to track the changes in the volume of the barium sulfate to show potential parenchymal or vascular lesions.

Additionally, the images were evaluated by nine independent examiners according to the following scoring system (Fig. 3):

0 = no lesion detectable

1 = minimal diffuse extravasation

2 = multiple diffuse extravasation or little extravasation at the exposed kidney pole

3 = massive/clear extravasation at the exposed kidney pole

The results were plotted against the shock wave energy. The examiners consist of three experienced urologists, three physicists and three computer scientists to show the validity of this scoring system independently of the medical background. The system and the individual scores were explained to all examiners.

## Results

### Fragmentation of BEGO-stones

Due to technical reasons with a discharge of the capacitor, the electrode had to be reset at times during the experiments. But we could demonstrate a fast pulverization of the BEGO stone. The finest fragmentation could be achieved using 18 and 20 kV voltage (Fig. 1). The number of shock waves and energy applied to the stone is shown in Table 1. There was no correlation between the number of shock waves and the powdering degree or the applied Energy (Joule) and the grade of pulverization of the stone. Fast pulverization could still be achieved with 100 Hz [15].

### Perfused porcine kidney

Basically, number of shock waves applied voltage and frequency had no direct correlation with the occurrence of parenchymal lesions. Figure 3 also shows an x-ray of a kidney with 20,000 shockwaves 100 Hz and 16 kV compared to 2000 SW 2 Hz and 24 kV. There is hardly any difference in vascular lesions (Grade 1-lesion). The detected lesions of the renal parenchyma were minimal, technical parameters had no significant impact and the lesions did not differ from the results of former experiments using 1–1.5 Hz in the same model [14, 16].

### Limitations

Because our study was performed in vitro, we cannot say how efficient stone fragmentation might be in vivo when more tissue surrounds the stone and kidney. On the other hand, it is unclear whether a parenchymal lesion might occur. These points need to be verified in further clinical studies.

## Discussion

There is no doubt, that the future of extracorporeal lithotripsy of urinary calculi very much depends on significant improvement in the performance of the lithotripter. This includes fine fragmentation of the calculus like endoscopic intrarenal lithotripsy in a reasonable time to minimize the need for secondary procedures. On the other side, the introduction of new concepts should not be associated with an increased risk of trauma to the kidney.

Recently the introduction of Burst wave lithotripsy (BWL) has gained significant interest. Early in-vitro studies demonstrated quick pulverization of different urinary stones, animal studies showed minimal lesions in the kidney, and first clinical trials were able to prove the efficacy of the principle [8–10, 17].

Interestingly, already in 1987 Dornier Medizintechnik GmbH published the first results on shock wave bursts [18]. At that time the shock wave application was triggered with the R-wave of the electrocardiogram. The idea was to apply shock wave bursts at a frequency of 100–200 Hz exactly after the R-peak of the ECG to minimize any impact on the heart stimulation with the patient in the water tank. Dornier stated, that based on their studies the efficacy of stone disintegration remains unchanged as do the lesions on the kidney and surrounding tissues. The main advantage should be the shortening of treatment time. They provided data, that such bursts could be best produced by electrohydraulic shock wave sources whereas piezo-electric systems would need a higher energy output. Electromagnetic sources are not suitable because the 10-to-20-fold higher output energy would be transformed completely into heat and would thermically burn the shock wave source (ie. flat coil).

On the other side, in 1988 Delius et al. published results on the effect of high-frequency shock waves. They used a 40 nF generator of the Dornier HM3 at 20 kV (3000 shocks) and compared the shock-wave induced renal trauma of 100 Hz versus 1 Hz in the model of a canine kidney [12]. Significantly more hemorrhages occurred in the kidney parenchyma if shock waves were administered at a rate of 100 waves per second. The authors argued against a direct shock wave effect and favored cavitation as the mechanism of shock wave damage although thermal effects could not be excluded. These results might be the reason, why Dornier did not continue the project of shock wave bursts.

In 1989, Vallancien et al. studied the use of frequencies between 1.25 and 10 Hz in an in-vitro model [19]. They found that for hard stones a lower rate produced better fragmentation than the fast rate. They concluded to use the lower frequency because it allowed treatment without analgesia.

Cavitation represents one of the most discussed physical phenomena associated with high-frequency application of shock waves and ultrasound waves. When applying high-frequency shock wave or burst wave technology, the size of the focal zone plays an important role, comparable to the situation in classic low frequency SWL [20]. Randad et al. achieved a 2.8-improved in-vitro fragmentation of 11 mm cylindric artificial stones by enlargement of the width of the focal zone of their BWL-source (350 kHz transducer) from 6 to 11 mm [21].

Persistence of cavitational microbubbles from one shock wave to the next can hinder the comminution process. Enhanced stone subdivision with decreasing rate of shock waves delivery has been demonstrated both in vitro [4] and in vivo [22]. These studies are corroborated by clinical trials in which higher success rates were observed for patients treated with low shock waves at a rate of 1 Hz in comparison to those at 2 Hz [23]. Such outcomes are demonstrative of the “shielding” phenomenon commonly associated with SWL, in which a population of prefocal cavitation nuclei—here, remnant bubbles created by the preceding low shock wave—act to attenuate the tensile component of the waveform and reduce the efficacy of cavitation at the stone surface.

In the presence of particulates released from stones, the positive pressure of the SW remained unaffected, but the trailing tensile phase of the pulse was significantly reduced at 120 SW/min. Cavitation bubbles do not persist between SWs. Thus, mature bubbles from one pulse do not interfere with the next pulse, even at 120 SW/min. However, cavitation nuclei carried by fine particles released from stones can persist between pulses. These nuclei have little effect on the compressive wave, but seed cavitation under the influence of the tensile wave. Bubble growth draws energy from the negative-pressure phase of the SW, reducing its amplitude. This likely affects the dynamics of cavitation bubble clusters at the stone surface, reducing the effectiveness of bubble action in stone comminution [24].

The recent study of Harper et al. using an ultrasound-based application of BWL already showed some limitations of this technology [17]. Only 19 of 41 patients could be included in this pilot study, mainly because the stone could not be seen by sonography or due to the obesity of the patients, the focal depth of the device was too short. In this context, high-frequency SWL using electrohydraulic technology could be easily integrated into a fluoroscopic system for stone localization and the focal depth of the device could be adjusted by change of the ellipsoid reflector. The high-frequency output depends only on the capacity of the generator.

## Conclusions

High-frequency shock wave lithotripsy can produce stone fragments small enough to spontaneously pass in a very short amount of time. The injury to the renal parenchyma is comparable to the results of the conventional SWL using 1–1.5 Hz. Further studies are needed to prove the concept in clinical cases.

## References

[1] Rassweiler J, Rieker P, Rassweiler-Seyfried MC (2020). Extracorporeal shock-wave lithotripsy: is it still valid in the era of robotic endourology? Can it be more efficient?. Curr Opin Urol.

[2] Knoll T, Alken P (2011). Beyond ESWL: new concepts for definitive stone removal. World J Urol.

[3] Miernik A, Wilhelm K, Ardelt P (2012). Modern urinary stone therapy: is the era of extracorporeal shock wave lithotripsy at an end?. Urologe A.

[4] Hein S, Miernik A, Wilhelm K (2016). Clinical significance of residual fragments in 2015: impact, detection, and how to avoid them. World J Urol.

[5] Rassweiler J, Rassweiler MC, Frede T, Alken P (2014). Extracorporeal shock wave lithotripsy: an opinion on its future. Indian J Urol.

[6] Koo V, Beattie I, Young M (2010). Improved cost-effectiveness and efficiency with a slower shockwave delivery rate. BJU Int.

[7] Maxwell AD, Wang YN, Kreider W (2019). Evaluation of renal stone comminution and injury by burst wave lithotripsy in a pig model. J Endourol.

[8] Ramesh S, Chen TT, Maxwell AD (2020). In vitro evaluation of urinary stone comminution with a clinical burst wave lithotripsy system. J Endourol.

[9] Zwaschka TA, Ahn JS, Cunitz BW (2018). Combined burst wave lithotripsy and ultrasonic propulsion for improved urinary stone fragmentation. J Endourol.

[10] Harper JD, Metzler I, Hall MK (2021). First in-human burst wave lithotripsy for kidney stone comminution: initial two case studies. J Endourol.

[11] Eisenmenger W (2001). The mechanisms of stone fragmentation in ESWL. Ultrasound Med Biol.

[12] Delius M, Mueller WG (1990). Biological effects of shock waves at a fast shock wave administration rate of fifteen Hertz. J Lithotr Stone Dis.

[13] Liu Y, Zhong P (2002). BegoStone–a new stone phantom for shock wave lithotripsy research. J Acoust Soc Am.

[14] Köhrmann KU, Bensemann J, Florian J (1994). The isolated perfused kidney of the pig: new model to evaluate shock wave-induced lesions. J Endourol.

[15] Wess OJ, Mayer J (2020). Fragmentation of brittle material by shock wave lithotripsy. Momentum transfer and inertia: a novel view on fragmentation mechanisms. Urolithiasis.

[16] Back W, Köhrmann KU, Bensemann J (1994). Histomorphologic and ultrastructural findings of shockwave-induced lesions in the isolated perfused kidney of the pig. J Endourol.

[17] Harper JD, Lingeman JE, Sweet RM (2022). Fragmentation of stones by burst wave lithotripsy in the first 19 humans. J Urol.

[18] Jocham D, Elff M, Müller C, Graff J, Sauerbruch TZR (1987). Dornier Lithotripters: HM3, HM4, HM5.

[19] Vallancien G, Munoz R, Borghi M (1989). Relationship between the frequency of piezoelectric shock waves and the quality of renal stone fragmentation. In vitro study and clinical implications. Eur Urol.

[20] Evan AP, McAteer JA, Connors BA (2008). Independent assessment of a wide-focus, low-pressure electromagnetic lithotripter: absence of renal bioeffects in the pig. BJU Int.

[21] Randad A, Ghanem MA, Bailey MR, Maxwell AD (2020). Design, fabrication, and characterization of broad beam transducers for fragmenting large renal calculi with burst wave lithotripsy. J Acoust Soc Am.

[22] Bohris C, Roosen A, Dickmann M (2012). Monitoring the coupling of the lithotripter therapy head with skin during routine shock wave lithotripsy with a surveillance camera. J Urol.

[23] Tailly GG, Tailly-Cusse MM (2014). Optical coupling control: an important step toward better shockwave lithotripsy. J Endourol.

[24] Bailey MR, Maxwell AD, Cao S (2022). Improving burst wave lithotripsy effectiveness for small stones and fragments by increasing frequency: theoretical modeling and ex vivo study. J Endourol.

